# Precision medicine for epilepsy: challenges and perspectives for an optimized clinical care pathway

**DOI:** 10.3389/fneur.2025.1644835

**Published:** 2025-09-11

**Authors:** Marcia Morita-Sherman, Eugen Trinka, Patrick Kwan, Akio Ikeda, Min Cho, Harald Hampel

**Affiliations:** ^1^Eisai Inc., Nutley, NJ, United States; ^2^Department of Neurology, Neurointensive Care, and Neurorehabilitation, Christian Doppler University Hospital, Paracelsus Medical University, Member of EpiCARE, Salzburg, Austria; ^3^Neuroscience Institute, Center for Cognitive Neuroscience, Christian Doppler University Hospital, Salzburg, Austria; ^4^Karl Landsteiner Institute for Clinical Neurosciences, Salzburg, Austria; ^5^Department of Neuroscience, School of Translational Medicine, Monash University, Melbourne, VIC, Australia; ^6^Department of Neurology, Alfred Health, Melbourne, VIC, Australia; ^7^Department of Neurology, Melbourne Brain Centre at Royal Melbourne Hospital, University of Melbourne, Melbourne, VIC, Australia; ^8^Department of Epilepsy, Movement Disorders and Physiology Kyoto University Graduate School of Medicine Shogoin, Kyoto, Japan

**Keywords:** precision medicine in epilepsy, systems medicine, biomarkers, digital technologies, data science

## Abstract

Epilepsies are a common, but heterogeneous group of brain disorders, characterized by an enduring predisposition to recurrent epileptic seizures. Recognizing epilepsies as a disease spectrum offers compelling opportunities to implement precision medicine in routine care. In this narrative review, we assess the status and development of precision epilepsy, compare its implementation with the advanced model of precision oncology, and discuss strategies to advance the implementation of precision medicine in epilepsy care. We aim to raise awareness about the current state-of-the-art approaches in precision epilepsy, emphasizing their potential to optimize epilepsy care. Rapid technological innovations provide the foundation to improve epilepsy research and management including the establishment of multi-dimensional biomarkers to aid disease subtyping and treatment decision. We also introduce emerging digital health technologies that will transform seizure monitoring and prediction. Advances in data science and artificial intelligence will deepen our knowledge of epilepsies, and may deconstruct and systematize historical, clinical, and descriptive concepts. Following a thorough examination of the current epilepsy landscape – including obstacles against precision medicine implementation and clinical adoption - we envision that the path toward precision epilepsy care lies in studies uncovering the mechanisms underlying systems-biology and neurophysiology-based epileptogenesis using technological innovations, such as genetic testing, fluid indicators, neuroimaging, neurophysiology, and wearable devices. We review the literature based on four core pillars - biomarkers, digital technologies, systems medicine, and data science - to pinpoint the unmet need for epilepsies and thus revolutionize disease management strategies.

## 1 Introduction

Epilepsies are a heterogeneous group of brain disorders (1), characterized by a continuing predisposition to epileptic seizures, with cognitive, psychological, and social consequences. Epilepsies impose a high burden on patients, caregivers, and society, disproportionately affecting low- and middle-income countries (2, 3).

Pharmacological treatment remains the primary method for achieving long-term seizure control despite limitations such as side effects and drug interactions (4). Existing challenges in epilepsy care include drug resistance, the lack of preventive or disease-modifying treatment (5, 6), and the absence of established biomarkers to predict seizure onset and treatment outcomes. Despite the ever-growing number of anti-seizure medications (ASMs) that target diverse molecular mechanisms, such as selectively modifying preictal-ictal transition currents, enhancing inhibition, blocking ionotropic glutamate receptors, and through interactions with elements of the synaptic release machinery; or by combinations of these mechanisms (7) approximately 30% of patients on ASM treatment continue to experience seizures, as observed over the past three decades (8, 9). Drug resistance remains a clinical challenge, largely due to an incomplete understanding of the underlying mechanisms of drug resistance. New treatment advances, such as the potential use of focused ultrasound to allow for selective blood–brain barrier permeability and localized drug delivery, hold promise for preventing seizures (10). Patients who fail to respond to treatment with ASM require a diagnostic re-assessment to confirm and refine the epilepsy diagnosis and should be evaluated for other treatment options, including surgical interventions, neuromodulation, and dietary therapies (4).

In the absence of disease-modifying treatments, a biology-based, holistic framework is needed to replace the current symptom-oriented care model (11, 12). The World Health Organization’s Intersectoral Global Action Plan (WHO-IGAP) on epilepsy aims to improve care in epilepsies by proposing strategies to reduce epilepsy stigma and burden, and promote research innovation (2, 3). The WHO-IGAP identifies precision medicine (PM) as a promising innovation with the potential to accelerate the improvement of epilepsy care (5, 13).

PM is defined as “prevention and treatment strategies that take the individual variability into account” and was proposed as a holistic framework to address the complexity of a multifactorial disease spectrum (14). Some treatment paradigms or decision-support tools have been inaccurately described as PM. This misinterpretation considers “precision” to mean localization, personalization, or prognostication. While these are components of a PM model for epilepsy, they do not encompass the full scope. PM is tailored to an individual’s specific multi-dimensional characteristics and consequently has the potential to revolutionize epilepsy care by improving early detection, diagnosis, classification, prevention, and treatment (13). While some use personalized medicine and PM interchangeably, they are not synonymous. Personalized medicine is a targeted approach that guides treatment decision-making based on evidence, individual conditions (genetic profile, environment, and lifestyle) and clinical skills of healthcare providers. The existing model of ASM selection is an example of personalized medicine. ASM selection considers clinical and socioeconomic factors and leverages evidence-based data, physician experiences, and patient preferences. Rather, PM is an extension of the traditional personalized care model by offering more precise and individualized care through the utilization of sophisticated diagnostic tools enabled by recent technological advances (13, 15).

Epilepsies provide compelling opportunities for PM given their measurable outcomes, known risk factors, and established animal models (16). To approximate and appreciate the special complexity of the human brain, affected by pathophysiological mechanisms leading to diverse clinical phenotypes, this review will explore PM in epilepsy using a previously established general PM framework for neurology and psychiatry (17) focusing on four main pillars: biomarkers, digital technologies, systems medicine, and data science, and will discuss major limitations to implementation compared to the ones faced by oncology. We highlight, based on examples, how the pillars of PM in epilepsy have already been established and how we can leverage the experience gained in oncology to further expedite the implementation of PM in epilepsy care. Ultimately, a better understanding of the current state of PM in epilepsy will illuminate the path forward. Finally, in this narrative review, we aim to raise awareness of the state-of-the-art in epilepsy care and how precision epilepsy can enhance the management of these complex diseases. While numerous examples are described in this review, providing a comprehensive overview of all tools currently available in epilepsy care is beyond the scope of this review.

## 2 PM in epilepsy: modeled from oncology

PM-oriented approach was pioneered and substantially advanced in oncology. Learnings from this development serves as a conceptual framework for other complex disease areas. Data extracted from tissue samples shared through collaborative studies led to the identification of specific gene alterations associated with many cancer types (18). The Cancer Genome Atlas (TCGA) collaborative research initiative (started in 2006) analyzed numerous cancer tissues and generated over 2.5 petabytes of genomic, epigenomic, transcriptomic, and proteomic data that is publicly available to researchers worldwide (19). TCGA played an essential catalytic and leadership role in oncology research.

The availability of large datasets from various omics levels enabled the use of computational tools, allowing an in-depth understanding of cancer at the system level. Challenges related to harmonization, volume, and complexity of data were, and still are, being addressed with modern data science techniques (19). This comprehensive genetic characterization of cancer informed the development of biomarkers and therapeutic targets, leading to the transition from a broad cytotoxic treatment to targeted therapies based on the molecular characteristics of individual tumors (18).

The successful integration of biomarkers, digital technologies, data science, and system biology principles shaped the landscape of PM in oncology (17). Following the success of PM in oncology, epilepsy research and clinical care took the first steps toward PM. Many conceptual and technical challenges faced by the epilepsy field coincide with those encountered by oncology.

The application of PM to epilepsy is limited by the complex pathophysiology and etiology of epilepsy and epileptogenesis and the interactions between pathological and physiological excitation and inhibition. Additionally, the dynamic nature of epileptiform activities over time, which includes both short-term fluctuations occurring within seconds to minutes around each seizure and the long-term changes that occur over several years can hinder the application of PM. Lastly, our limited understanding of the interactions among mechanisms at various scales—ranging from cell intrinsic changes to multiple cell types and their interaction among one another, and local neural-glial and long distance neural networks that involve multiple brain regions—adds complexity to the implementation of PM in epilepsy (20). Unlike oncology, where identified biomarkers have a direct relationship to the pathogenic mechanisms of cancer, in epilepsy, biomarkers may represent epiphenomena or be confused with the etiology of the diverse underlying mechanisms and different diseases causing epilepsies.

It is crucial to recognize these differences as they relate to our foundational understanding of epilepsy mechanisms that the pillars of precision epilepsy stand upon and not allow these complexities to hinder our progress in advancing the field.

## 3 The pillars of pm in epilepsy

### 3.1 Biomarkers

Biomarkers are indicators of physiological and pathogenic processes or responses to an exposure or intervention (12, 21). Biomarker categories include susceptibility, diagnostic, monitoring, prognostic, predictive, pharmacodynamic treatment/response, and safety (Table 1) (21). In oncology, biomarkers such as genetic profiling and liquid biopsies revolutionized treatment decisions (22, 23). Continued advances in the development, validation, and application of diverse biomarkers are pivotal in PM (12, 24), as they bridge the gap between disease pathophysiology and clinical care.

**Table 1:** Examples of different categories of biomarkers available in epilepsy care.

Category	Examples
Susceptibility/risk	Epileptiform abnormality as a susceptibility/risk biomarker for seizure recurrence after a single unprovoked seizure
Diagnostic	Genetic testing (e.g., *SCN1A* mutations as a diagnostic biomarker for Dravet syndrome, Neuroimaging tools, Multimodal imaging co-registration tools, Automated detection of brain abnormalities in MRI, Quantitative evaluation of Neuroimaging, invasive and non-invasive EEG data, Detection of autoantibodies in autoimmune epilepsies).
Monitoring	Blood levels of lamotrigine as a monitoring biomarker to adjust treatment dose during pregnancy, continuous EEG in status epilepticus, seizure detection devices.
Prognostic	MRI as a monitoring biomarker for patients with brain tumors
Predictive	Neurophysiological testing as a predictive biomarker to identify patients at risk for memory and language deficits following epilepsy surgery, multimodal imaging co-registration tools (the comparison between functional MRI results and structural abnormalities facilitates the prediction of potential postoperative deficits)
Pharmacodynamic response	EEG patterns as a pharmacodynamic biomarker to assess response to status epilepticus treatment
Safety	Human leukocyte antigen (HLA-B*15:02) allele as a genetic biomarker to identify patients at risk for hypersensitivity to carbamazepine

EEG, electroencephalography; HLA, human leukocyte antigen; MRI, magnetic resonance imaging.

An integrated multi-modality biomarker approach is crucial for the integrative holistic conception of epilepsy (24). Biomarkers, based on genetic, neuroimaging, electrophysiology, and body fluids assessments, were already incorporated into epilepsy clinical practice. We provide examples of biomarkers currently being used toward a more “personalized and sub-population tailored” clinical approach and discuss their growing role in the clinical implementation of PM. Integrating data from various modalities will enhance our understanding of the complexities of epilepsy in a more holistic way, allowing us to move from personalized clinical care to a PM approach involving individual level differences. We do not intend, however, to provide a comprehensive review of all available biomarkers currently being used and studied in epilepsies.

#### 3.1.1 Genetic biomarkers

About 70 to 80% of epilepsies are estimated to have genetic variants underlying the disease (25). Clinical tests using comprehensive gene panels, exomes, or genomes (26) led to the identification of hundreds of genes associated with monogenic and polygenic epilepsies (27). These mutations affect the function of ion channels, neurotransmitter receptors, and other molecular components leading to seizures (Box 1).

Box 1 **Key take away points.**

- Epilepsies provide compelling opportunities for PM given their heterogeneity, measurable outcomes, known risk factors, and established animal models.
- Advances in biomarkers, digital technologies, systems medicine, and data science are the key pillars of implementing PM in epilepsy.
- By comparing the oncology and epilepsy fields, we can gain insights into implementing PM in epilepsy care successfully.
- The path toward PM in epilepsy care lies in studies aimed at understanding systems-biology epileptogenesis using technological innovations.
- PM has the potential to optimize epilepsy care in the predictive, preventive, personalized, and participatory dimensions as envisioned by the P4 paradigm.

The role of genetics in epilepsies is more complex than in other diseases since a similar clinical phenotype may originate from different genetic mechanisms (e.g., in West syndrome) (27, 28) and mutations in one gene can be associated with a wide range of phenotypes (e.g., in epilepsies mediated by mutations in the SCN2A gene) (26). Despite this high genetic heterogeneity and pleiotropy, significant strides were made to uncover novel genes associated with epilepsies (18).

Elucidating genetic profiles in epilepsy clinical practice advanced diagnosis, the development of tailored treatments, prediction of ASM response, drug metabolism, and risk of adverse events. The continued development and characterization of genetic models of epilepsies (*in vivo* and *in vitro*) can inform us about relevant epilepsy pathways and additional points of intervention.

Dravet syndrome (DS) is an example of PM being applied in epilepsy clinical care (Box 2). DS is an infantile-onset developmental and epileptic encephalopathy associated with pathogenic variants in the SCN1A gene, which encodes a subunit of a sodium channel in the brain (29, 30). A deeper understanding of the disease at a molecular level and the availability of confirmatory genetic biomarkers revolutionized the management of DS. A precise diagnosis offers the opportunity to customize treatment by avoiding ASMs with sodium channel blocking property that may worsen seizures and negatively impact cognitive development. It also allows genetic and prognosis counseling and enables approaches to prevent status epilepticus by providing guidance on antipyretics, vaccinations, and rescue medication (30).

Box 2 **Precision medicine in Dravet syndrome.**

Our understanding of Dravet syndrome (DS) has gone from zero to a detailed, yet individualized, comprehension of its mechanism in less than 50 years (31). Clinically, DS is characterized by the intractable, prolonged seizures alongside fevers and cognitive impairment (32, 33). DS onset peaks in the first year of life. The association between DS and de novo pathogenic variants in the SCN1a gene was first reported in 2001 and has been repeatedly confirmed since then (32). Studies on how SCN1a mutations affect the structure and activity of the sodium channels enhanced our understanding of DS (33). Various SCN1a alterations correspond with different levels of loss-of-function, and less frequently, gain-of-function mutations in the sodium channel, explaining the wide variability in DS severity and therapeutic response to antiseizure medications (29, 34). Mutations on the SCN1A gene are often *de novo* and constitutional; however, they can also be inherited from a parent with a less severe phenotype or present as somatic mosaicism (33), further highlighting the complexity of genetics in epilepsies. This mechanistic understanding of DS allowed the establishment of animal models with specific mutations and thus offered opportunities for targeted treatments. Even though there is still a lot to be researched in DS, the acquired knowledge on the pathophysiology of DS, its complexity, and individual variability has already led to innovations in prevention and treatment strategies. The future of PM in epilepsy will likely follow a similar path (i.e., a new finding of the pathophysiological mechanism will lead to follow-up questions, which will eventually contribute to the decoding of epilepsy etiology).

Identifying SCN1A mutations also has implications for developing new treatments, with some drugs being tested and approved specifically for DS (30). It also allowed the development of disease-modifying therapies, such as genetic therapies and antisense oligonucleotides that restore the sodium channel, some of which are already being tested in clinical trials (30). DS exemplifies how genetic biomarkers can lead to a systems medicine level understanding of epilepsy pathology and transform clinical care. A PM approach to DS encompassing changes from genetic mutations to protein expression, channel functions, and environmental influences, has significantly altered how we comprehend, manage, and develop new treatments for patients with DS.

Genetic biomarkers in epilepsy can also be used to tailor treatment selection in clinical practice. For instance, the human leukocyte antigen (HLA-B*15:02) allele was found to be highly associated with carbamazepine-induced severe skin reactions in broad Asian populations. Carrying out HLA-B*15:02 allele genotyping helps guide treatment selection by identifying individuals with an increased risk of severe skin reactions to carbamazepine (35, 36).

Despite the high genetic heterogeneity and pleiotropy observed in epilepsies, similarly to oncology, genetic will play a major role toward advancing PM in epilepsy.

#### 3.1.2 Neuroimaging biomarkers

Epilepsy surgery is one of the most compelling examples of how a multi-modal approach can improve epilepsy care. To achieve successful surgical outcomes and preserve eloquent brain areas, it is imperative to accurately identify the epileptogenic zone. Structural, functional and metabolic neuroimaging biomarkers including magnetic resonance imaging (MRI), fluorodeoxyglucose positron emission tomography (FDG-PET), and single-photon emission computerized tomography (SPECT), are often part of the epilepsy pre-surgical evaluations (12). The concordance of multimodality data, along with safety considerations, is critical in defining the extent of the surgical resection in epilepsy (37).

Technological advances have facilitated the interpretation and integration of multimodal data. For example, post-processing tools are used to geometrically align images so that corresponding pixels/voxels represent the same structure (38). Continued improvements in co-registration tools have allowed their use in epilepsy surgery evaluations enabling physicians to overlay images from different modalities to facilitate the evaluation of concordance between finding from different modalities. Co-registration tools also offer a unique opportunity to evaluate cross-sectional or longitudinal data with a high degree of precision (39).

The incorporation of new MRI sequences, such as arterial spin labeling, into presurgical planning can enhance patient prognostication beyond structural imaging (40). In addition, the use of translational molecular imaging with its variety of molecular probes [e.g., translocator protein 18 kDa (TSPO) PET ligands] has the potential to be leveraged for diagnostic imaging, patient stratification, therapy monitoring, and drug development as described in other pathologies (41). Improvements in imaging quality, such as higher field MRI, combined with machine learning (ML) tools applied to MRI have shown compelling results in detecting subtle malformations of cortical development (42, 43), while automatic quantitative assessments have enabled more accurate evaluations of structural abnormalities (39). Some of these tools are already available in clinical practice.

In epilepsies, certain features and patterns extracted from brain images can serve as biomarkers, providing valuable information about structure, function, and connectivity. Following the same path as oncology, neuroimaging tools can be utilized not only to define etiology but also hold significant potential to serve as biomarkers for PM care.

#### 3.1.3 Electrophysiological biomarkers

Analyses of ictal and interictal electroencephalogram (EEG) patterns are critical for defining surgical care, diagnosis, treatment, and prognosis in epilepsies (37). EEG can access brain activity either in an invasive or non-invasive manner. Advances in invasive EEG monitoring exemplify how PM can be applied to epilepsy clinical practice. The development of a tailored electrode placement map, based on the convergence of multimodal assessment data, is crucial to successfully delineate the epileptogenic zone in surgical cases (37). By co-registering CT and MRI, physicians can visualize the precise location of the invasive electrode, thus facilitating the interpretation of invasive EEG data and allowing comparison of EEG results with the location of other structural and metabolic findings. Additional tools, including magnetoencephalography (MEG), electric, and magnetic source imaging will further improve epilepsy evaluation (44). In epilepsy clinical practice and research, improvements in the automatic analysis of EEG patterns and seizure detection are also providing valuable information about brain activity with the potential to advance precision epilepsy further. For instance, mathematical modeling has identified additional EEG patterns such as infra-slow (e.g., ictal Direct Current (DC) shifts, red slow) and high-frequency oscillations, which has refined the localization of the epileptogenic zone (45–47). In the field of seizure forecasting, data acquired from recording devices, such as deep brain stimulation (DBS) or responsive neurostimulation (RNS) devices, and the development of long-term EEG devices will enable novel biomarkers for a seizure forecasting (48). The detection of critical slowing down preceding seizures suggests that slow waves could be used as indicators in seizure forecast algorithms (49). In addition, studies on seizure distribution following the circadian, multiday, or seasonal rhythms further demonstrated the potential use of EEG data to predict seizure recurrence, especially when combined with other physiological measures and environmental influences (48). Continued advancement in the automated detection of EEG patterns hold significant potential for improving the field.

Over the past decade, lags in the analysis of EEG recordings come from the large data volume, confining the application in clinical practice. New tools, such as ML and mathematical models, will enable a broader use of EEG as a biomarker (50–52).

#### 3.1.4 Bio fluids biomarkers

Body fluid matrices are attractive because they can illustrate biological changes in the brain while circumventing the blood–brain barrier and are a relatively inexpensive and minimally invasive source of additional biomarkers. Variations in inflammatory cytokines, redox states, hormone levels, and signaling pathways are correlated with neurologic dysfunction (12, 53).

In epilepsy research, there is a need to explore the use of fluid biomarkers (CSF and serum) in clinical care further. For example, high levels of neurofilament light chain (NfL) in status epilepticus represent a promising biomarker of seizure-related neuronal damage (54). Increased levels of certain proteins, such as S100B and glial fibrillary acidic protein (GFAP), in the blood or cerebrospinal fluid, may indicate neuronal damage and could be associated with epilepsy severity (55).

Advances in the field of autoimmune epilepsies exemplify the role of fluid biomarkers in PM and how they can transform clinical practice. The detection of autoantibodies in some types of epilepsies (such as the anti-NMDA receptor antibodies) allows classification of autoimmune epilepsies as clinically independent entities that can be managed with tailored treatments targeting the autoimmune response (56).

### 3.2 Digital technologies in precision epilepsy

Digital technology is defined as systems that use computing platforms, connectivity, software, and/or sensors for healthcare and related uses (57). There is rapid growth of clinical data in electronic health records and other health-related information databases via digital technologies, such as wearable devices, smartphones, and edge computing (17).

In oncology, digital solutions that have furthered PM include digital patient records, digital pathology, digital platforms for clinical trials, and the ability to share data digitally among healthcare providers and institutions. The same tools are available in the epilepsy field and are essential to foster collaboration between centers. In epilepsies, there are multiple applications of digital tools for PM, ranging from facilitating sharing of research data to developing devices to aid patient care.

Seizure detection devices exemplify how digital technologies can play a role in PM for epilepsy care and research. Generalized tonic–clonic seizures and focal to bilateral tonic–clonic seizures can now be accurately detected by wearable devices, lowering risk for injuries and sudden death (58, 59). Wearable devices for seizure detection were created based on the insights from the dynamics and rhythms of ictal and interictal activities acquired from EEG data (58) combined with known motor and autonomic features of seizures. Data collected by wearable devices, such as surface electrocardiogram, electromyography, and wrist accelerometer, allowed further advances in automated seizure detection (59). Commercially available video monitoring and infrared-based devices also have the potential to be used for seizure detection but need further development (60). Seizure detection devices can also increase accurate measurements of seizure outcomes (e.g., seizure frequency and duration); this information has typically been collected via patient-reported data, which can be inaccurate and biased (13, 61). Since epilepsy treatment is based mainly on seizure outcomes, this lack of precision directly affects clinical management.

Despite encouraging advances in the field, the sensitivity of seizure-detection devices is paramount to optimizing epilepsy management. False alarms, particularly for seizures without convulsions, can have a detrimental impact on patients with epilepsy and their caregivers. Well-designed studies are warranted to evaluate the effectiveness, usability, adoption, and clinical impact of these devices (59, 62). Digital devices also allow the collection of a diverse repertoire of disease-related phenotypes in a convenient, unobtrusive, and longitudinal manner (17) providing an opportunity to further advance the understanding of epilepsies, seizure patterns, and triggers.

Digital biomarkers in epilepsy have the potential to address several challenges in the field that significantly impact treatment decisions, including seizure prediction, detection, and quantification. The ongoing development and adoption of digital tools will continue to shape the landscape of PM, fostering collaborations and research breakthroughs.

### 3.3 Systems medicine

Systems medicine is a translational extension of systems biology. Systems biology refers to an integrative research approach designed to address the complexity of biological systems (63). The main principle of systems biology is that a simultaneous multitude of molecular interactions from various levels occurring at any one time, are combined in a holistic manner to produce a phenotype. A systems biology approach uses computational and mathematical tools to elucidate causative dynamics, intermediate endophenotypes, and/or clinical features of a condition (17, 63, 64).

In oncology, the systems biology approach adopting integrative omics enabled a deeper understanding of disease pathophysiology by taking into consideration genetic profiling, molecular interactions, signaling pathways, and regulatory networks (18). The addition of “omics” to a term implies a comprehensive, or global, assessment of a set of molecules or components within a particular biological system (65). Advances in omics technologies allowed researchers to integrate information from different levels (genetic, transcriptomic, proteomic, and metabolic) to build comprehensive models of biological systems (66). Systems biology provides the analytical and computational framework to integrate, interpret, and model omics data, enhancing our understanding of normal *versus* pathologic conditions (63). Insights gained from systems biology have furthered systems medicine in oncology by informing the development of new diagnostic and therapeutic strategies.

Like its application in oncology, systems medicine has the potential to decipher the complexity underlying epilepsies. Even though we have an incomplete understanding of many epilepsy syndromes at the systems level, there are some examples of how systems medicine has advanced epilepsy care.

Epilepsy associated with tuberous sclerosis complex (TSC) represents a unique model of disease that exemplifies how system medicine can improve care. TSC is a rare multisystem genetic disorder characterized by the development of tumors and caused by mutations in TSC1 or TSC2 genes, leading to dysregulation of the mammalian target of rapamycin (mTOR) pathway. Gene mutations in TSC have downstream effects on neurobiological systems in a dynamic and complex way that results in considerable phenotypic variability (67) with different degrees of developmental disabilities and seizure burden (67, 68). In-vitro and in-vivo studies have undercover the roles of TSC1 and TSC2 genes, which include tumor suppression, neuronal network development, morphology and function, oxidative stress, inflammation, and regulation of specific microRNAs in the neurological environment (67).

A system medicine approach continues to contribute to advances in the development of biomarkers and therapies for TSC and has led to improved targeted treatments (e.g., mTOR inhibitors and gene therapy), tailored ASM selection, and advances in epilepsy surgery (67). A recent trial showed that preventive treatment of TSC with ASM can modify the natural history of seizures (69), further emphasizing how PM has the potential to change epilepsy care.

By integrating various types of biological data and employing computational models, systems medicine will allow us to further understand and incorporate the heterogeneity and multi-factorial nature of epilepsy toward clinical care.

### 3.4 Data science in precision epilepsy

Data science is an interdisciplinary field that encompasses a range of techniques, including statistical analysis, ML, and data visualization, to uncover patterns, trends, and relationships in complex datasets from various domains (70).

Advances in biomarkers and digital health technology and the creation of epilepsy consortia, databases, and repositories generated a massive amount of data (12, 16, 71, 72). Despite being a challenge for traditional statistical approaches, the availability of comprehensive datasets provided an opportunity to incorporate complexity and use a system medicine approach to further our understanding of epilepsies.

Innovative analytical tools based on artificial intelligence (AI) are rapidly taking on a leading role in all fields of science and society and have the potential to revolutionize epilepsy research. For example, ML can identify patterns hidden in large-scale unprocessed data using supervised or unsupervised models (17, 71, 72). ML tools to predict drug treatment or surgical outcomes, or automatically detect MRI abnormalities are already being developed for epilepsy care (73). Deep learning, a subset of ML, employes multi-layered neuronal networks to analyze complex patterns, enabling the interpretation of data features and relationships (17). Unsupervised deep learning can reveal the pathological heterogeneity underlying epilepsies by clustering high-dimensional data and has recently been employed to classify EEG patterns for epilepsies (74, 75). Other computing innovations such as molecular dynamics modeling, whole-cell simulation, tailored animal models, and “digital twin,” provide a glimpse into the potential clinical applications of AI-based PM in epilepsy research (76–78).

As data availability progressively increases, the limited power of classical computing will significantly hinder the analysis of large datasets in epilepsy research (79). Quantum computing provides a potential solution with the ability to handle complex analytical demands in shortened times (80). Enhanced ML models allow integration of sparse and noisy data to overcome limitations imposed by traditional approaches. Interpretability of findings, validity of data outputs, and ethical concerns are some barriers to implementation of AI in epilepsy and in the healthcare industry. Together, data science and AI will broaden the range of research tools to better pinpoint the mechanisms underlying epileptogenesis, and help develop disease-modifying, individualized treatments (13).

## 4 Discussion

As illustrated by the examples above, the pillars of PM in epilepsy have already been established. The convergence and integration of the pillars of PM will forge a robust foundation, enabling the field to thrive and advance. By examining the incorporation of PM in epilepsy, we can identify variances and parallels between oncology and epilepsy and gain insights on how to overcome barriers and successfully implement PM in epilepsy care.

Following the knowledge acquired from precision oncology, there is a need to further integrate data from different disease measurement modalities and shift from the single-center study paradigm to a large interdisciplinary multi-center collaborative effort to fully understand epilepsies. To break down information silos and foster deeper collaboration, updates on health information policies and laws, and the development of clear rules for collaboration are needed. Concerns related to data sharing can be overcome by improvements in de-identification technology, data homogenization, and the secure transfer of medical data or federated learning (12, 19, 81). Further advancements and data integration across the pillars of PM in epilepsy are crucial for its full implementation.

Incorporating PM in epilepsy presents a set of unique challenges. In contrast to oncology, where abnormal tissues and cells are accessible for analysis, in epilepsies, such histological data is accessible in only a small proportion of epilepsy cases. Furthermore, while oncology treatment outcomes can be objectively measured by tumor growth and mortality, epilepsy outcomes rely mostly on seizure frequency, a subjective measurement. Outcomes based on seizure-count are not always precise due to the episodic nature of seizures, the reliance on the caregiver’s description, and the wide variability of seizure patterns (61). Advances in digital technologies and biomarkers can help overcome this barrier.

**Figure 1:**
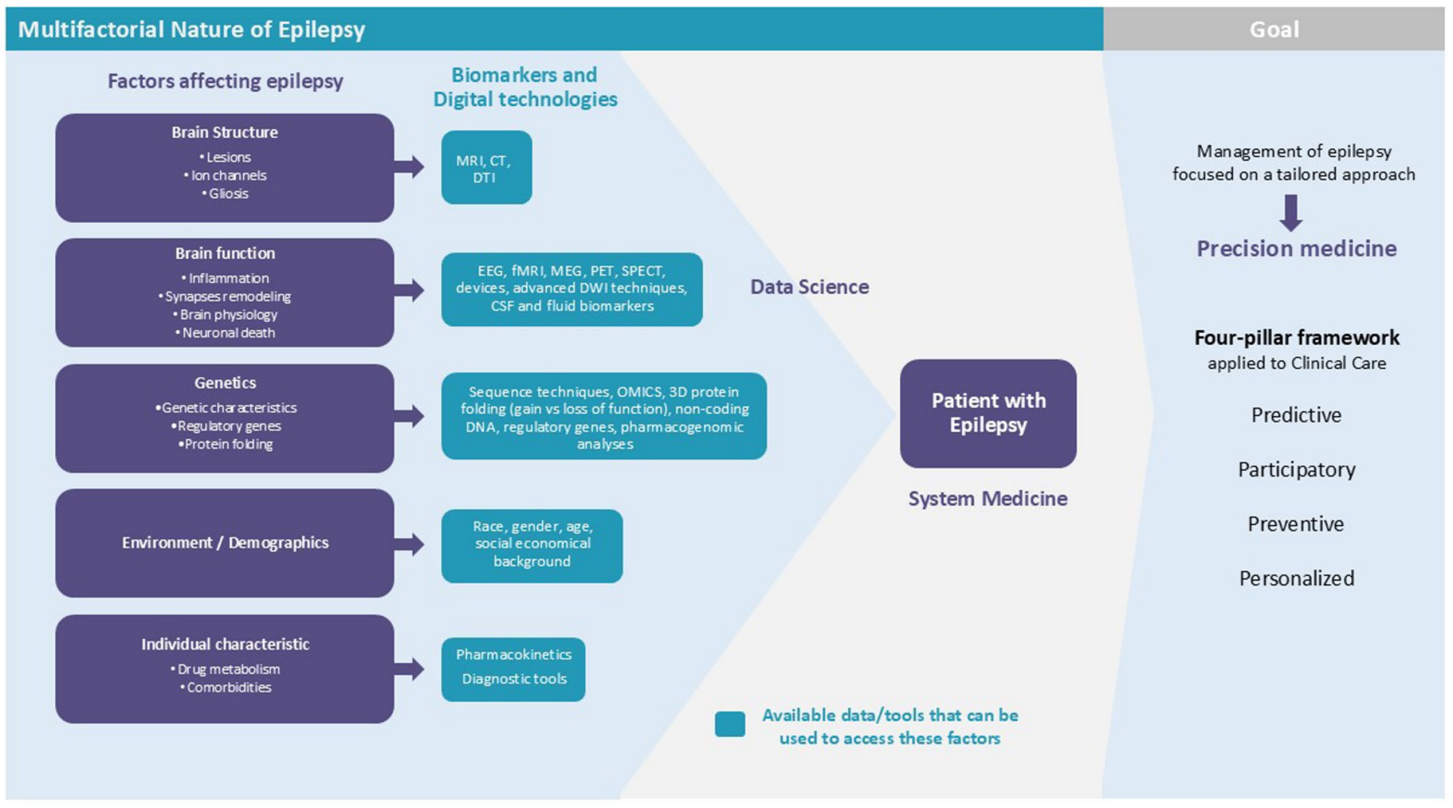
The shifting landscape toward precision epilepsy care. Epilepsies are a group of biologically complex and heterogeneous disorders. With the availability of novel biomarkers and digital tools, combined with advances in computational and mathematical tools (data science), there is an opportunity to integrate and analyze multimodal data at all levels. The systems medicine approach offers great potential to overcome the complexity of phenotypic heterogeneity and the multifactorial nature of epilepsy. A holistic understanding of epilepsies will ultimately lead to optimized clinical care, moving us toward precision medicine and allowing us to fully implement the P4 paradigm: predictive, preventive, participatory, and personalized care. CT, computed tomography; DTI, diffusion tensor imaging; EEG, electroencephalography. fMRI, functional magnetic resonance imaging; MRI, magnetic resonance imaging; PET, positron emission tomography; SPECT, single-photon emission computerized tomography.

Differently from oncology, epilepsies are a group of biologically complex, heterogeneous central nervous system disorders that can occur due to an extensive range of potentially overlapping etiologies (e.g., genetic, brain insults, metabolic, immune, and idiopathic). Seizures can occur as the main feature of an epilepsy syndrome (e.g., juvenile myoclonic epilepsy), or they can be a component of a more complex disease (e.g., developmental and epileptic encephalopathies) (82, 83). Additionally, epilepsies frequently co-occur with other conditions, such as mood disorders, sleep disturbances, and cognitive impairments (84). In other words, epilepsy is not a single disease, but a spectrum of disorders characterized by recurrent seizures, further emphasizing the extent of its complexity. The gradual evolution of PM will likely follow a self-fueled trajectory characterized by continuous integration between the four pillars and a dynamic and interconnected network in which advancements in one area contribute to improvement in others. As the field matures, these feedback loops and interdependencies are expected to drive continuous progress, making PM an increasingly powerful and integral part of healthcare. This integrated framework has the potential to gradually optimize clinical care in the predictive, preventive, personalized, and participatory dimensions across a variety of epilepsy syndromes as envisioned by the P4 paradigm (85) (Figure 1).

Several factors will foster further development of PM to improve the quality of care in epilepsies, with education and participation of healthcare providers being critical to this endeavor. For the full implementation of PM, health information policies and laws must be updated to ensure the safe use of AI across healthcare sectors and provide appropriate insurance coverage for broad access to biomarker tests and digital health services. Ultimately, the goal of PM is to help patients with epilepsy achieve improved clinical outcomes and reduced socioeconomic burden.

According to the Gartner Hype Cycle, emerging technologies follow five phases: technology trigger, peak of inflated expectation, trough of disillusionment, slope of enlightenment, and plateau of productivity (13). We are hopefully moving toward the slope of enlightenment to bring PM in epilepsy to fruition soon.

## 5 Conclusion

This narrative review provides an overview of recent achievements and existing challenges for the implementation of PM in epilepsy. In both epilepsy and oncology fields, the availability of data collected from validated biomarkers and digital technologies can inform the system medicine approach and, with innovations in the data science field, advance the development of PM-based clinical care. The complexity and diversity of epilepsy pathophysiology, challenges in outcome measurements, and the diversity of treatment options (leading to a lack of abnormal tissue samples for analysis) contribute to the delayed implementation of PM in comparison to oncology.

To advance PM in epilepsy, we need to shift from the traditional clinical phenotype-oriented mindset to a biomarker-guided multimodal and clinical-biological model encompassing the multifactorial nature of epilepsy across stages. New exploratory, hypothesis-independent (multi-) omics technologies combined with computational (multi-modal) neuroimaging and neurophysiology advances can identify and validate additional biomarkers and harness the advantages offered by systems medicine to help implement PM-based prevention, early detection, diagnosis, classification, and treatment, resembling the progress observed in oncology.

## Glossary

AI: Artificial intelligence
ASM: Anti-seizure medication
DBS: Deep brain stimulation
DC: Direct Current
DS: Dravet syndrome
EEG: Electroencephalogram
FDG-PET: Fluorodeoxyglucose positron emission tomography
GFAP: Glial fibrillary acidic protein
HLA: Human leukocyte antigen
MEG: Magnetoencephalography
ML: Machine learning
MRI: Magnetic resonance imaging
mTOR: mechanistic target of rapamycin
NfL: Neurofilament light chain
PM: Precision medicine
RNS: Responsive neurostimulation
SPECT: Single-photon emission computerized tomography
TCGA: The Cancer Genome Atlas
TSC: Tuberous sclerosis complex
TSPO: Translocator protein 18 kDa
WHO-IGAP: World Health Organization’s Intersectoral Global Action Plan
